# The Potential Therapeutic Efficacy of Lactobacillus GG in Children with Food Allergies

**DOI:** 10.3390/ph5060655

**Published:** 2012-06-19

**Authors:** Roberto Berni Canani, Margherita Di Costanzo, Vincenza Pezzella, Linda Cosenza, Viviana Granata, Gianluca Terrin, Rita Nocerino

**Affiliations:** 1 Department of Pediatrics, University of Naples “Federico II”, 80131, Naples, Italy; Email: mara.dicostanzo@live.it (M.D.C.); cinzia3006@gmail.com (V.P.); lindacosenza@libero.it (L.C.); vivigranata@hotmail.com (V.G.); ritanocerino@alice.it (R.N.); 2 European Laboratory for the Investigation of Food Induced Diseases, University of Naples “Federico II”, 80131, Naples, Italy; 3 Department of Woman’s Health and Territorial Medicine, University of Rome “La Sapienza”, Rome, Italy; Email: gianluca.terrin@uniroma1.it (G.T.)

**Keywords:** food allergy, probiotic, intestinal microflora, immune system, *Lactobacillus* GG, cow’s milk allergy, tolerance acquisition, atopic march, pediatrics, infants

## Abstract

Food allergy (FA) continues to be a growing health concern for infants living in Western countries. The long-term prognosis for the majority of affected infants is good, with 80–90% naturally acquiring tolerance by the age of five years. However, recent studies suggest that the natural history of FA is changing, with an increasing persistence until later ages. The pathogenesis of FA as well as oral tolerance is complex and not completely known, although numerous studies implicate gut-associated immunity and enteric microflora, and it has been suggested that an altered composition of intestinal microflora results in an unbalanced local and systemic immune response to food allergens. In addition, there are qualitative and quantitative differences in the composition of gut microbiota between patients affected by FA and healthy infants. These findings prompted the concept that specific beneficial bacteria from the human intestinal microflora, designated probiotics, could restore intestinal homeostasis and prevent or alleviate allergy, at least in part by interacting with the intestinal immune cells.

## 1. Introduction

Food allergy (FA) is a major health issue in Western countries, where some evidence has suggested that the prevalence of the disorder in childhood has increased in recent years. Although any food can provoke a reaction, relatively few foods are responsible for the vast majority of significant food-induced allergic reactions in children: Cow’s milk, hen’s eggs, soy, wheat, fish, peanuts, and shellfish [1]. Cow’s milk allergy (CMA) is the most common FA in early childhood, with an estimated incidence ranging between 2% and 3% in infants and marginally lower in older children [2]. The majority of children will regain tolerance to cow’s milk proteins (CMP) within the first 5 years of life [3]. However, recent studies suggest that the natural history of CMA is changing, with an increasing persistence until later ages [4,5]. At present, the only proven treatment consists of elimination of CMP from a child’s diet. For infants this requires the substitution of standard infant formulas with a hypoallergenic formula [6]. The intestinal microflora plays a crucial role in the establishment of tolerance to food antigens. Differences in terms of microflora genus and species have been observed between allergic versus healthy individuals in some studies. Epidemiological data show that allergic children have higher levels of Clostridia, and lower levels of Bifidobacteria*.* Nevertheless, Bifidobacteria and Lactobacilli are found more commonly in the composition of the intestinal microflora of non-allergic children [7]. The enhanced presence of these probiotic bacteria in the intestinal microflora seems to correlate with protection against atopy [8]. Modifications to intestinal microflora in allergic individuals through the use of food supplements such as probiotics or prebiotics have been proposed as a strategy to treat or prevent allergic diseases. Probiotic research and industry have continued to grow from early observations, and the global sales of probiotic ingredients, supplements, and foods amounted to $21.6 billion in 2010 and are expected to reach $31.1 billion by 2015 with an annual growth rate of 7.6% for the next 5-year period [9]. However, the growth of the probiotic industry has not been paralleled by advances in basic probiotic research and clinical trials determining their efficacy. We have only begun to understand the mechanisms and limitations of probiotics, which vary greatly among the strains and among the treated individuals [10]. Moreover, many clinical studies yielded negative results, leading to very polarized views among scientists and clinicians on the usefulness of probiotics in general populations and in specific disorders. With the aim to help physicians in the use of probiotics, the purpose of this paper is to review the use of probiotics, in particular of *Lactobacillus rhamnosus* GG (LGG), in CMA and to summarize what is currently know about its health benefits as dietary supplements added to food products marketed for children, such as infant formulas.

## 2. Rationale for the Use of Infant Formulas Containing Probiotics in Cow’s Milk Allergy

Microbial gut colonization begins after birth and this process is affected by the newborn infant’s gestational age, mode of delivery and diet. The colonizing bacteria originate mainly from the mother’s gut and vaginal tract [11]. After delivery, breast feeding continues to enhance the original inoculum by the introduction of specific lactic acid bacteria, Bifidobacteria and other bacteria from the mother’s skin. These bacteria set the basis for intestinal microflora development and modulation. Early exposure to commensal bacteria plays a crucial role in Th1/Th2 polarization and proper immune regulatory mechanisms. Toward the end of the first month of life in developing countries, the most common species in breast-fed infants are Bifidobacteria and Lactobacilli, whereas formula-fed infants have equal colonization with Bacteroides and Bifidobacteria species [12]. As stated above, Lactobacilli and Bifidobacteria are found more commonly in the composition of the intestinal microflora of non-allergic children and the enhanced presence of these probiotic bacteria in the intestinal microflora seems to correlate with protection against atopy [8]. In contrast, epidemiological data have shown that atopic children have a different intestinal microflora from that of healthy children, with higher levels of Clostridia and lower levels of Bifidobacteria. Furthermore, other studies have also shown that early colonization with potentially more pathogenic bacteria such as *Clostridium difficile* and *Staphylococcus aureus* is more likely to occur in children who go on to develop allergies [13]. These data suggest the rationale for the administration of probiotics to infants at risk of atopic diseases, particularly for those who are formula-fed. In addition, probiotics might also be considered for treatment of subjects already suffering from CMA on the basis of their multiple mechanisms of action that occur within the intestinal lumen or within and beyond the intestinal mucosa. The potential mechanisms of action of LGG against CMA are certainly multiple and exerted at different levels (Table 1). Local influences of probiotics potentially include: Hydrolysis of antigenic peptides in the gut lumen, modulation of intestinal permeability and reduction of systemic penetration of antigens, increased local IgA production and modulation of local inflammation, stimulation of epithelial cell growth and differentiation [14,15,16]. Some possible systemic effects consist of anti-inflammatory effects mediated by toll like receptors (TLRs), Th1 skewing of responses to allergens and activation of tolerogenic dendritic cells (DCs), in addition to T regulatory cells production, and tolerance acquisition [7,17]. Emerging evidences suggests that many effects elicited by probiotics are dependent on epigenetic modulation of gene expression. These effects could be important during critical periods of early development. Epigenetic mechanisms regulates both Th1 and Th2 differentiation and changes in promoter methylation are a prerequisite for FoxP3 expression and Treg differentiation [18,19].

**Table 1:** Schematic representation of the potential mechanisms of action of probiotics in children with cow’s milk allergy.

Effects within intestinal lumen	Effects at mucosal level	Effects beyond the intestinal mucosa
modulation of intestinal microflora	modulation of intestinal permeability	modulation of innate/adaptive immune system
hydrolysis of antigenic peptides	stimulation of cell growth and differentiation	induction of oral tolerance
		impact on the enteric nervous system

## 3. Oral Tolerance and Intestinal Microflora

**Figure 1:**
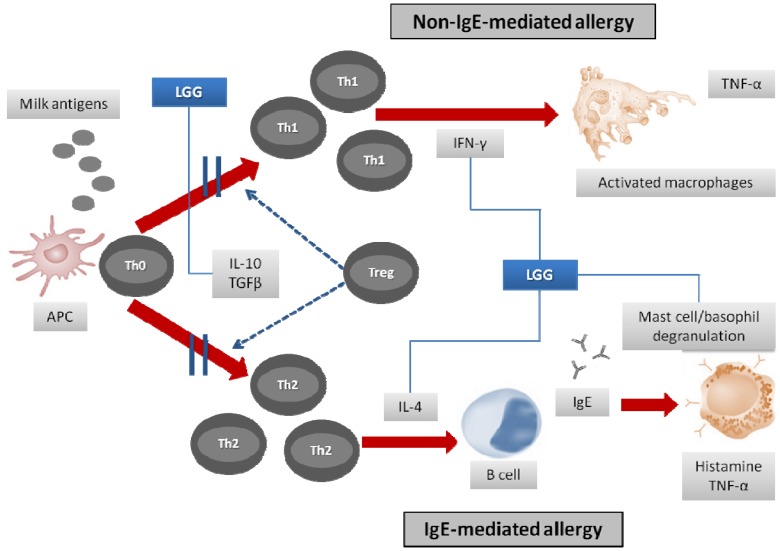
Lactobacillus GG acts on different mechanisms of CMA.

Food antigens and intestinal microflora constitute the majority of the antigen load in the intestine, and the “default” reaction of the immune system confronted with them leads to systemic unresponsiveness. This phenomenon is known as oral tolerance and is a key feature of intestinal immunity [20]. The complex interaction between intestinal contents and immune and non-immune cells result in an environment that favors the tolerance by the induction of IgA antibodies and CD4 + T regulatory cells (producing IL-10 and IFN-γ) [17]. This ensures that a homeostatic balance is maintained between the intestinal immune system and its antigen load, so that it retains the ability to recognize dangerous and harmless antigens as foreign and preserves the integrity of the intestinal mucosa. The inappropriate immune responses to food and intestinal microflora that are responsible for FA are a result of a deregulation of these crucial processes [21]. An allergic reaction mainly corresponds to the activation of Th2 cells against food allergens and occurs in two phases: The first phase corresponds to transport of the allergen through the intestinal barrier, its capture by antigen presenting cells (DCs or enterocyte), and its presentation to naive Th0 cells, which differentiate in the presence of IL-4 into Th2 cells. Activated Th2 cells then produce an IL-4 cytokine that enables the production of allergen-specific IgE by B cells [22]. These secreted IgEs then bind to mast cells via the IgE receptor FcεRI. The activation phase corresponds to the degranulation of mast cells after further exposure to the same allergen that links directly with specific IgE on the surface of these cells. This phenomenon triggers a release of the allergic mediators involved in clinical manifestations of allergy. In addition to acute allergic reactions triggered by IgE-mediated immune responses to food proteins, there are cell-mediated manifestations (Figure 1).

There is mounting evidence that the gut microbiota acquired during the early post natal period is required for the development of oral tolerance. Oral tolerance induction to dietary antigen is not possible in germ-free mice. However, a reconstitution of the gut microbiota of germ-free mice with *Bifidobacterium infantis* during the neonatal period, but not later, restored the susceptibility to oral tolerance induction [23]. Moreover, a transplanted healthy infant microbiota had a protective impact on sensitization and CMA in mice [24].

### 3.1. Symptoms’ Resolution

The first objective in the treatment of CMA is symptoms’ resolution. At present, the only proven treatment consists of elimination of CMP from the child’s diet. For infants this requires the substitution of standard infant formulas with a hypoallergenic formula. In the last years, numerous studies have been performed to test the potential effects of various strains of probiotic bacteria in the treatment of allergies. In this context, one of the most extensively studied probiotic bacteria has been LGG [25]. The studies with this strain suggested a therapeutic effect in patients with atopic eczema. Atopic eczema is a frequent manifestation of FA [26]. Administration of LGG to highly selected food-allergic patients (age < 2 years, challenge-proven and mild-to-moderate eczema) improved the eczema score significantly [27]. Studies in infants with eczema who received formulas supplemented with LGG have shown benefit in decreasing gastrointestinal symptoms [28]. For instance, after a challenge study in infants allergic to CMP, fecal IgA levels were detected to be higher and TNF-α levels were lower in LGG applied group compared to the placebo [29]. Nermes *et al*. investigated the interaction of LGG with skin and gut microbiota and humoral immunity in infants with atopic dermatitis. This study showed a statistically significant decrease of IgA- and IgM-secreting cells 1 month after starting an intervention with extensively hydrolyzed casein formula supplemented with LGG. This might indirectly indicate that LGG enhances gut barrier function and accelerates immunological maturation in infants with atopic dermatitis. Especially the finding of significant increase in memory B cells in LGG treated infants could be of particular importance [30]. Moreover, LGG induced IFN-γ secretion in infants with CMA and in infants with IgE-associated dermatitis, but interestingly, not in infants with no CMA. This supports the view that the pattern of intestinal microflora may be aberrant in infants with an atopic predisposition, and the beneficial effects of probiotics are evident only in this group [31]. The addition of LGG to an extensively hydrolyzed casein formula significantly improved the recovery of the inflamed colonic mucosa compared with extensively hydrolyzed casein formula alone in infants with blood in the stools and presumptive cow’s milk allergic colitis, as indicated indirectly by greater decreases in fecal calprotectin and in the number of infants with persistence of occult blood in stools after 1 month [32].

### 3.2. Tolerance Acquisition

**Figure 2:**
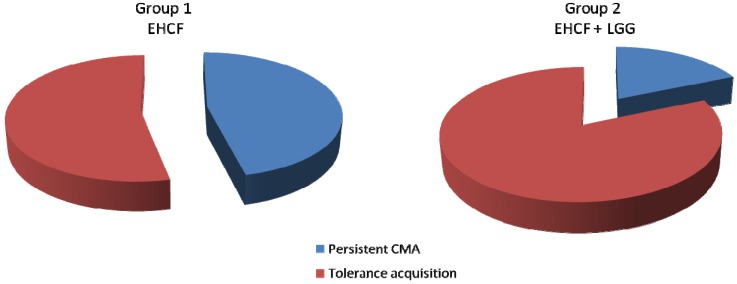
Lactobacillus GG accelerates tolerance acquisition in infants with cow’s milk allergy. Rate of patients with documented tolerance acquisition at the double blind placebo controlled food challenge after 12 months of diet therapy with an extensively hydrolyzed casein formula (EHCF) or with an extensively hydrolyzed casein formula containing LGG (EHCF+LGG) (modified from reference [34]).

The second objective in the treatment of CMA is tolerance acquisition. The majority of children will regain tolerance to CMP within the first 5 years of life. However, recent studies suggest that the natural history of FA is changing, with an increasing persistence until later ages. Hol *et al.* evaluated the effect of probiotic supplementation on the acquisition of tolerance toward cow’s milk in infants. They concluded that supplementation of extensively hydrolyzed formula with a combination of 2 probiotics, *Lactobacillus casei* CRL431 and *Bifidobacterium lactis* Bb-12, does not accelerate CM tolerance in infants with CMA [33]. In contrast, we recently demonstrated that an extensively hydrolyzed casein formula containing LGG was able to accelerate the development of tolerance acquisition in infants affected by IgE- or non-IgE-mediated CMA. Infants (aged 1–12 months), consecutively referred for strongly suspected CMA but still receiving CMP, were invited to participate in the study. Subjects were randomly allocated to one of the two groups of dietary interventions: Group 1, received an extensively hydrolyzed casein formula; and group 2, received an extensively hydrolyzed casein formula containing LGG (at least 1.4x10^7^ CFU/100 mL) (figure 2). After 6 and 12 months, full clinical evaluation, skin-prick tests (SPTs), atopy patch tests (APTs), and double-blind, placebo-controlled food challenges (DBPCFCs) were planned. At the end of the study period, DBPCFC resulted negative in 15 of 28 infants in the control group (53.6%) and in 22 of 27 in infants receiving the extensively hydrolyzed casein formula containing LGG (81.5%, p = .027). The acquisition of clinical tolerance was confirmed by the negative response of SPTs and APTs showed by all patients with negative DBPCFC. In addition, all subjects consumed regular doses of cow’s milk daily without showing any signs or symptoms related to CMA for 6 months after the negative DBPCFC result, which is compatible with the persistence of clinical tolerance.

These findings suggest a disrupting approach for infants affected by CMA, namely an “active dietotherapy” able to reduce the time of tolerance acquisition [34].

### 3.3. Prevention of Atopic March and Intestinal Functional Disorders

Cow’s milk allergy is often the first manifestation of the so-called "atopic march", characterized by the appearance of other allergic manifestations in later years, such as allergic rhinitis and asthma. It appears to be caused by a regional allergic response with breakdown of the local epithelial barrier that initiates systemic allergic inflammation. Genetic and environmental factors predispose to developing the allergy. Thus, a potential third objective in the treatment of a child with CMA could be the prevention of the atopic march. There are data to support four possible interventions to prevent the allergic march from progressing to asthma: Supplements of dietary probiotics, exclusive breast feeding during the first few months of life, or, alternatively use of extensively hydrolyzed infant formulas, treatment with inhalant allergen immunotherapy by either subcutaneous or sublingual methods [35]. In this regard, it is important for future studies to also examine the long term effects of the addition of LGG to an extensively hydrolyzed casein formula on atopic march. Moreover, CMA constitutes a risk factor for the development of functional gastrointestinal disorders in children [36] and the administration of a probiotic strain with proved efficacy could constitute an easy and acceptable method to prevent functional gastrointestinal diseases in children with CMA. Probiotic bacteria can impact on enteric nervous system and modulate intestinal pain through the induction of opioid and cannabinoid receptors [37]. In particular, a study by Gawronska *et al.* suggests that LGG may be useful in children with irritable bowel syndrome [37,38].

## 4. Safety of LGG Added to Infant Formula

The addition of probiotics in formulas used for the management of CMA requires that they be proven safe and are well tolerated. LGG has over 25 years of safe use, including administration to preterm infants. According to the European Society of Pediatric Gastroenterology and Nutrition (ESPGHAN) and the American Academy of Pediatrics, a formula must be tested in a properly designed DBPCFC and can be considered hypoallergenic when demonstrated with 95% confidence that at least 90% of infants and children with confirmed CMA would have no reaction to the formula under double-blind, placebo-controlled conditions. Recently, Muraro *et al.* demonstrated that an extensively hydrolyzed casein formula remains hypoallergenic following the addition of LGG, satisfying both the ESPGHAN and American Academy of Pediatrics guidelines [39].

## 5. Conclusions

Numerous data suggest gut microbiota as a potential target to prevent or treat food allergies, and in particular CMA. These findings prompted the concept that specific beneficial bacteria from the human intestinal microflora, designated probiotics, could restore immune system homeostasis in children with CMA. *Lactobacillus* GG is the single probiotic with the greatest number of *in vitro* and *in vivo* evidences on possible effects in pediatric allergic disorders. The mechanism of these beneficial effects are multiple, ranging from modulation of intestinal microflora composition, to direct effect on intestinal mucosa structure and function, and on local and systemic immune response. The recent evidence on the beneficial effects elicited by LGG in children with CMA support a possible “nutritional immunology approach” in these patients able not only to efficiently cure the symptoms but also to accelerate tolerance acquisition. Larger trials are needed to confirm these findings, to better define the mechanisms of action and to evaluate the potential factors influencing the response in subjects with CMA.
